# Sutureless aortic valve replacement in Takayasu arteritis: A case report

**DOI:** 10.1016/j.xjtc.2026.102260

**Published:** 2026-01-19

**Authors:** Masashi Bungo, Hisashi Uemura, Yuji Sakashita, Yoshio Teshima, Ken-ichi Watanabe, Mitsuhiro Yamamura, Taichi Sakaguchi

**Affiliations:** Department of Cardiovascular Surgery, Hyogo Medical University, Nishinomiya City, Hyogo, Japan

**Figure:**
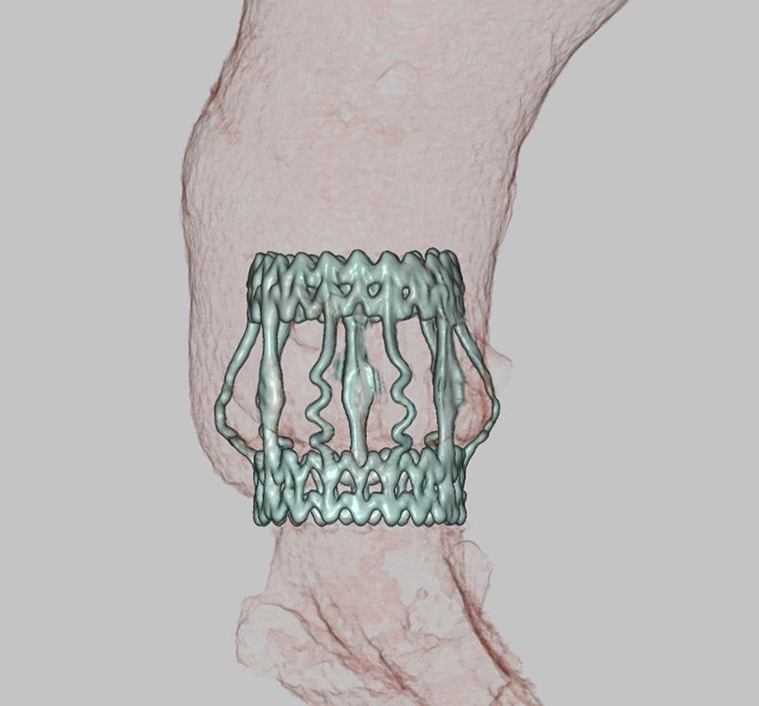
Self-expanding Perceval valve replaces aortic valve in Takayasu arteritis.

**Central Message**

Sutureless aortic valve replacement using a Perceval valve provides a safe and feasible option for Takayasu arteritis treatment by minimizing suture stress on fragile aortic tissue.

Aortitis syndrome is a rare, chronic systemic vasculitis of unknown etiology that involves the aorta, its major branches, the aortic valve, and the pulmonary artery. Among its subtypes, Takayasu arteritis is associated with aortic regurgitation (AR) in approximately 13% to 44% of cases.^1^ Surgical aortic valve replacement (AVR) in these patients is challenging because the inflamed aortic tissues are fragile, which increases the risk of prosthetic valve detachment and pseudoaneurysm. Here, we report successful management of severe AR in a patient with Takayasu arteritis using a sutureless Perceval valve (CORCYM SRL, previously LivaNova). Institutional review board approval was not required. Written informed consent for publication was obtained from the patient.

## Case Report

A 59-year-old man was urgently admitted to our hospital for general fatigue and exertional dyspnea. The patient had previously been monitored for severe AR at our institution but had discontinued treatment 2 years earlier. On admission, a systolic blood pressure difference >20 mm Hg between the upper limbs was observed, and given the AR history of the patient, aortitis syndrome was considered.

**Figure 1:**
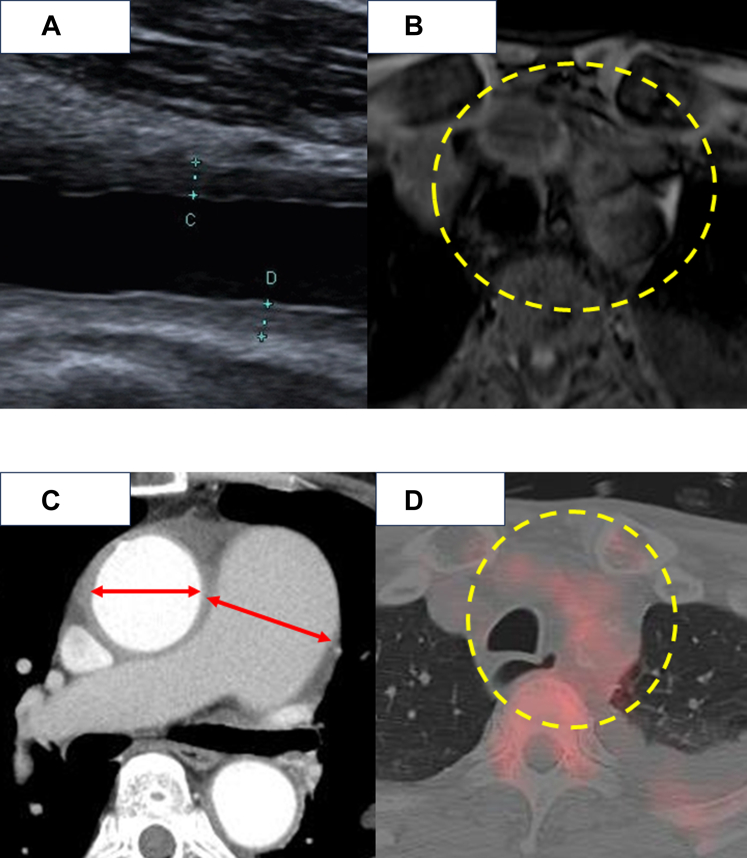
A, Carotid ultrasonography showing the characteristic “macaroni sign” in the common carotid artery, indicative of concentric wall thickening, typical of Takayasu arteritis. B, Contrast-enhanced magnetic resonance imaging demonstrating circumferential wall thickening of the cervical trifurcation arteries (highlighted by a *yellow circle*). C, Contrast-enhanced computed tomography (CT) revealing dilatation of the ascending aorta and the main pulmonary artery (*red arrows*). D, Positron emission tomography–CT showing mild radiotracer uptake in the same arterial segments (*yellow circle*), consistent with inflammatory activity.

Carotid ultrasonography revealed the macaroni sign in the common carotid artery (Figure 1, *A*). Contrast-enhanced magnetic resonance imaging showed circumferential wall thickening of the aorta and the 3 branch vessels of the aortic arch (Figure 1, *B*). Additional imaging findings on contrast-enhanced computed tomography (CT) included dilatation of the ascending aorta (maximum diameter: 43 mm; Figure 1, *C*) and main pulmonary artery (maximum diameter: 47 mm; Figure 1). Preoperative CT showed an annular diameter of 28 mm, a sinus of Valsalva diameter of 39 mm, and a sinotubular junction diameter of 42 mm. On the basis of 2022 American College of Rheumatology/European Alliance of Associations for Rheumatology classification criteria for Takayasu arteritis,^2^ Takayasu arteritis was diagnosed.

Laboratory tests on admission showed an elevated erythrocyte sedimentation rate (104 mm/h), whereas positron emission tomography−CT demonstrated mild radiotracer uptake in the aorta and cervical branch vessels (Figure 1, *D*), suggesting high disease activity. To minimize the risk of postoperative anastomotic pseudoaneurysm or prosthetic valve dehiscence, surgical intervention was deferred until inflammatory marker normalization.

The patient was started on 30 mg of glucocorticoids, which was gradually tapered to 5 mg over approximately 3 weeks. However, during administration of 5 mg, C-reactive protein levels increased again, prompting the addition of 6 mg of methotrexate. After this, C-reactive protein levels normalized, and AVR was performed approximately y weeks after treatment initiation for Takayasu arteritis.

Considering the risk of mediastinitis attributed to immunosuppressive and glucocorticoid therapy, surgery was performed via a right minithoracotomy through the third intercostal space. Cardiopulmonary bypass was initiated through cannulation of the right femoral artery and vein. Intraoperative inspection revealed a dilated ascending aorta with wall thickening and inflammatory changes of the adventitia. Dense adhesions were present between the ascending aorta and surrounding structures, which were carefully dissected. The ascending aorta was clamped, and a transverse aortotomy was made. Selective antegrade cardioplegia administration achieved adequate cardiac arrest. The native aortic valve exhibited a normal, tricuspid structure.

Intraoperative sizing demonstrated that the native aortic annulus exceeded 27 mm, which was above the recommended upper limit for the Perceval XL prosthesis. Therefore, symmetric pledgeted commissuroplasty was performed at the left–right and left–noncoronary commissures using nonabsorbable sutures to achieve uniform annular downsizing. Repeated sizing confirmed appropriate size, and the Perceval XL valve was subsequently implanted. The aortotomy was closed in 2 layers and reinforced externally with felt strips (Video 1).

Histopathologic examination revealed marked thickening of the intima, accompanied by lymphocytic infiltration in the adventitia, confirming the diagnosis of Takayasu arteritis. Glucocorticoid therapy was resumed postoperatively, and inflammatory markers were monitored.

**Figure 2:**
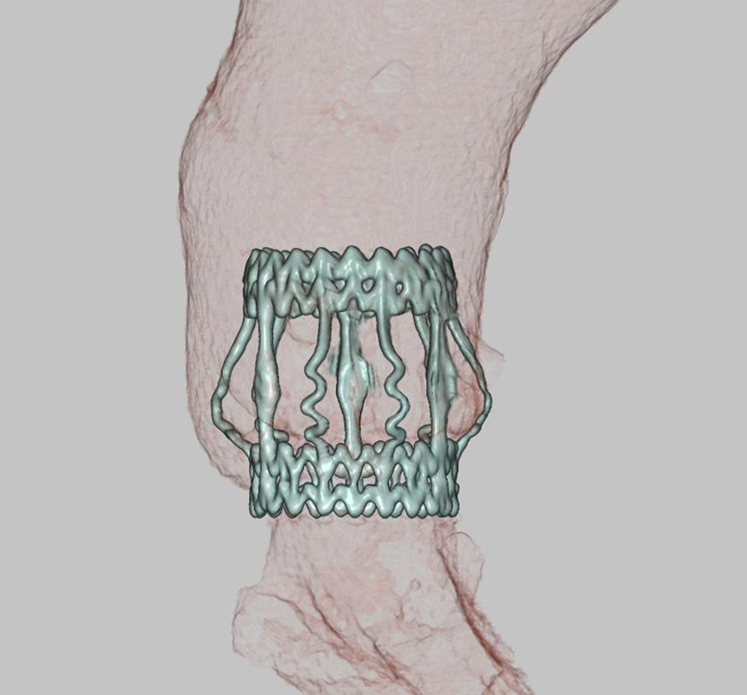
Postoperative 3-dimensional computed tomography demonstrating no evidence of an anastomotic pseudoaneurysm and confirming that the Perceval valve remained securely in position without evidence of migration.

The postoperative course was clinically uneventful. However, transient hepatobiliary enzyme elevation was observed on postoperative day 12, postponing patient discharge. The patient was discharged in stable condition on postoperative day 17. Follow-up included blood tests and transthoracic echocardiography at 6- to 23-month intervals, with contrast-enhanced CT performed when clinically indicated. At the 18-month follow-up, no evidence of anastomotic pseudoaneurysm, bioprosthetic valve dysfunction, or paravalvular leakage was observed (Figure 2).

## Discussion

AR in Takayasu arteritis is primarily caused by annular dilatation secondary to ascending aortic inflammation, accompanied by impaired leaflet coaptation from fibrotic contracture. Although valve repair was considered, inflammatory involvement of the annulus and leaflets, together with limited leaflet geometric height, substantially increased the risk of recurrent regurgitation; therefore, valve replacement was selected in this case.

Surgical AVR in this setting is technically challenging and produces suboptimal clinical outcomes. A Japanese multicenter study reported a 15-year survival of 76% after AVR, with nearly 10% of cases resulting in prosthetic valve detachment or graft failure.^3^ Moreover, surgical intervention during active disease resulted in a 7-fold increase in postoperative complications over the first 5 years compared with procedures performed after adequate inflammatory control.^4^ Our findings highlight the importance of surgical strategy and timing in this patient population.

Sutureless valves may prove advantageous in this context because they enable suturing on inflamed and fragile annular tissue, reducing the risks of future prosthetic valve dehiscence. Sutureless valves have yielded favorable results in other inflammatory conditions, such as prosthetic valve endocarditis, further supporting their use with fragile aortic tissue.^5^ The Perceval valve incorporates a self-expanding nitinol stent that conforms to the annulus. This unique design may also enable the valve to tolerate mild postoperative annular dilation, a concern particularly relevant to Takayasu arteritis. As the radial force generated by the Perceval valve is weaker than that of transcatheter valves, it is unlikely to promote progressive annular dilatation. Nevertheless, the long-term impact of chronic radial force on an inflamed or previously dilated annulus remains uncertain, and careful long-term follow-up is required.

Previous reports have suggested that paravalvular leakage and prosthetic valve dehiscence after AVR in inflammatory aortopathy are more frequently observed when using everting suture techniques. In the present case, this technique was deliberately avoided to minimize localized stress on the fragile annular tissue.

Careful aortic root anatomy evaluation is critical when considering indications for using the Perceval valve in patients with aortopathy. The manufacturer provides aortic dimension recommendations; however, the ascending aortic diameter alone should not be considered a definitive exclusion criterion for Perceval valve implantation. In clinical practice, annular size, sinotubular junction morphology, and overall aortic root anatomy are often more relevant, and these factors guided decision-making in the present case. Here, the aortic root showed moderate, symmetric dilatation and appeared suitable for implantation of the Perceval valve, as confirmed by postoperative CT (Figure 2).

To the best of our knowledge, this is the first report of sutureless Perceval valve implantation for Takayasu arteritis–related AR, demonstrating the feasibility and short-term efficacy of this approach. However, long-term outcomes remain unknown and further studies are required to fully assess the Perceval valve's benefits and durability.

## Conflict of Interest Statement

The authors reported no conflicts of interest.

The *Journal* policy requires editors and reviewers to disclose conflicts of interest and to decline handling or reviewing manuscripts for which they may have a conflict of interest. The editors and reviewers of this article have no conflicts of interest.

## Supplementary Data

Video 1 Intraoperative view showing the implantation of a Perceval sutureless valve. The video demonstrates annular sizing, positioning, and deployment sequence. Video available at: https://www.jtcvs.org/article/S2666-2507(26)00067-2/fulltext.
