# Supplementary figures and images for: Sutureless aortic valve replacement in Takayasu arteritis: A case report
Source: JTCVS Tech. 2026 Jan 19;36:102260. doi: 10.1016/j.xjtc.2026.102260 (PMC13069545; doi:10.1016/j.xjtc.2026.102260)

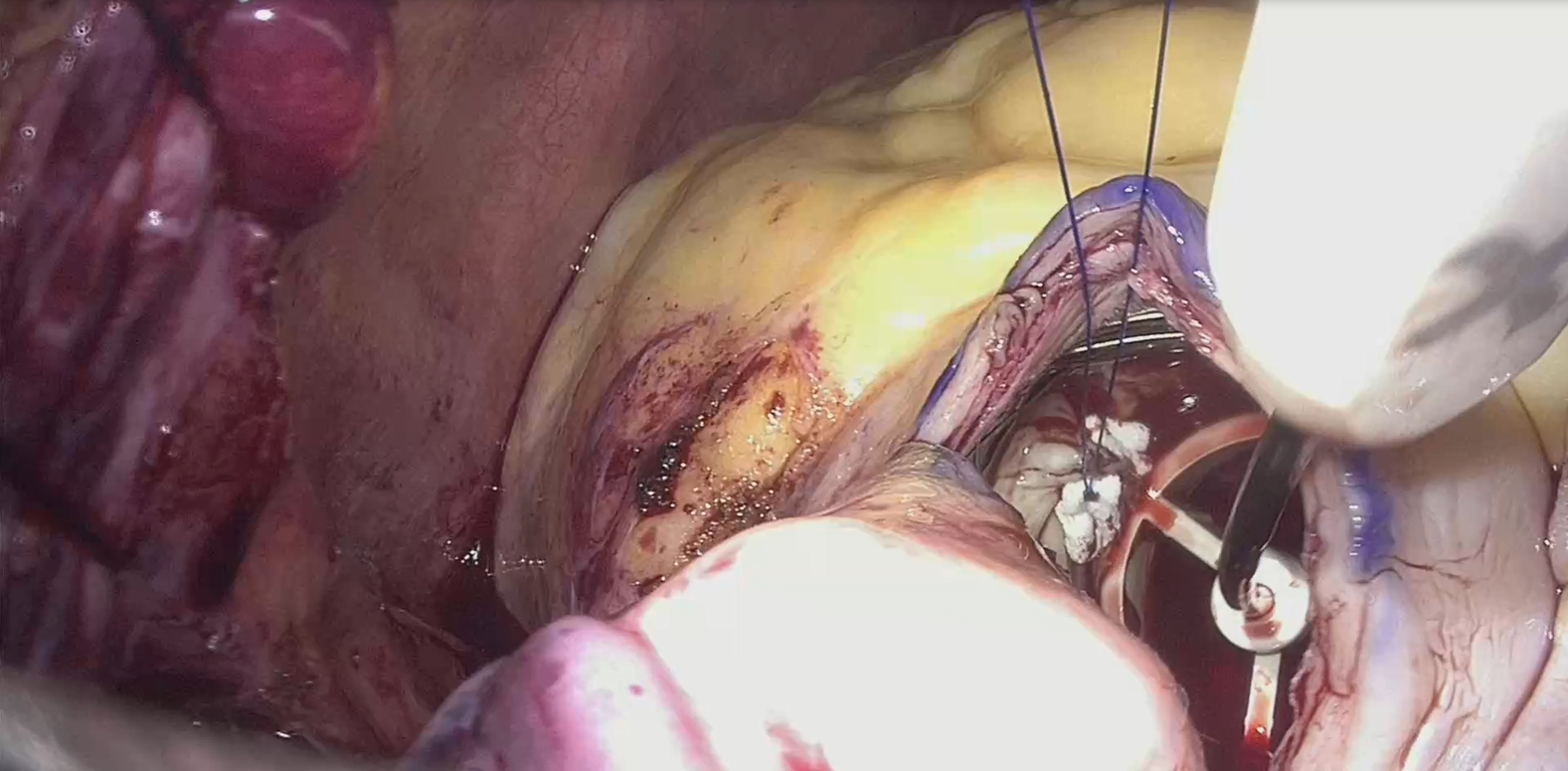

Supplement: Video 1 — Intraoperative view showing the implantation of a Perceval sutureless valve. The video demonstrates annular sizing, positioning, and deployment sequence. Video available at: https://www.jtcvs.org/article/S2666-2507(26)00067-2/fulltext. [file fx2.jpg]
